# Nicking Activity of M13 Bacteriophage Protein 2

**DOI:** 10.3390/ijms26020789

**Published:** 2025-01-18

**Authors:** Esma Aybakan, Tanil Kocagoz, Ozge Can

**Affiliations:** 1 Department of Medical Biotechnology, Institute of Health Sciences, Acibadem Mehmet Ali Aydinlar University, 34752 Istanbul, Türkiye; esmaaybakan@hotmail.com; 2 Department of Medical Microbiology, School of Medicine, Acibadem Mehmet Ali Aydinlar University, 34752 Istanbul, Türkiye; 3 Department of Biomedical Engineering, Faculty of Engineering and Natural Sciences, Acibadem Mehmet Ali Aydinlar University, 34752 Istanbul, Türkiye; ozge.can@acibadem.edu.tr

**Keywords:** M13 bacteriophage p2, nicking enzyme, nicking reaction, P2 cloning, single-strand DNA viruses

## Abstract

Gene II Protein (Gp2/P2) is a nicking enzyme of the M13 bacteriophage that plays a role in the DNA replication of the viral genome. P2 recognizes a specific sequence at the f1 replication origin and nicks one of the strands and starts replication. This study was conducted to address the limitations of previous experiments, improve methodologies, and precisely determine the biochemical activity conditions of the P2 enzyme in vitro. For these purposes, the gene encoding P2 was cloned in *Escherichia coli* and expressed as a hybrid protein together with a green fluorescent protein (P2-GFP). P2-GFP was purified via metal affinity chromatography, and its nicking activity was determined by conversion of supercoiled DNA to open circular or linear forms. We discovered that, among the two loops of the f1 origin defined previously, P2 can recognize just the A1 loop. When a supercoiled plasmid containing the f1 origin was treated with P2-GFP, the plasmid was present in an open circular form, indicating that a nick was created on only one of the strands. However, when the A1 loop sequence was inserted into the 3′ ends of both strands by cloning a PCR product obtained by primers with the A1 loop sequence, the plasmid was linearized by treatment with P2-GFP, indicating that nicks were created on both strands. Certain infectious diseases are caused by single-stranded DNA viruses, and some of them have specific nicking enzymes that enable strand displacement and free 3′ end of a single strand that works as a primer for their replication mechanisms like M13 bacteriophages, such as parvovirus B19. Despite there being different host viruses such as bacteria and humans, their DNA replication mechanisms are very similar in this concept. Investigating the features of the P2-nicking enzyme may deepen the understanding of human pathogenic single-stranded viruses and facilitate the development of drugs that inhibit viral replication.

## 1. Introduction

**Figure 1:**
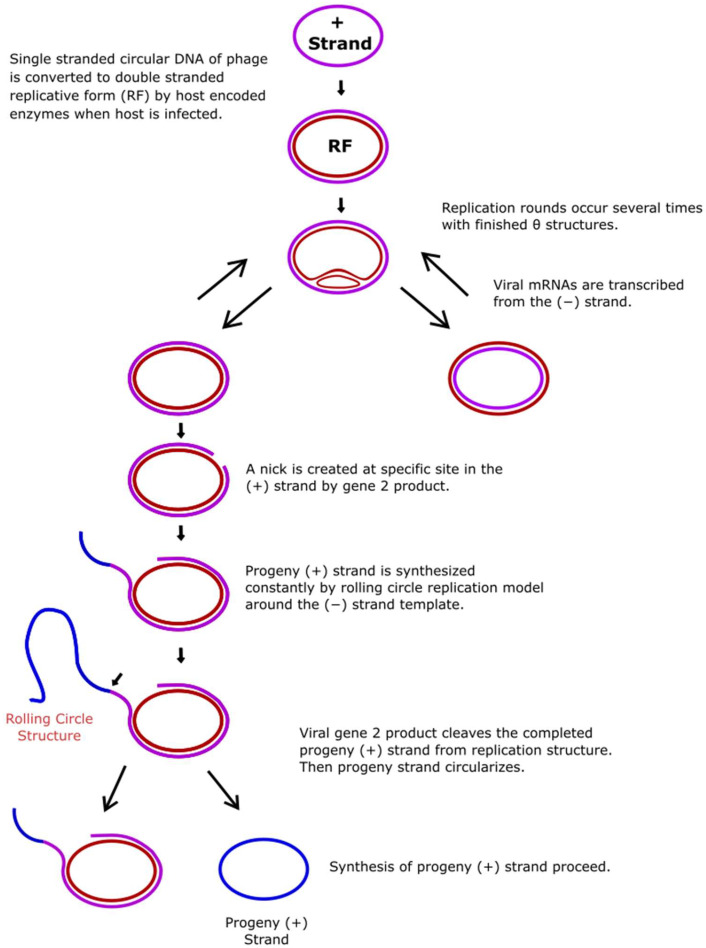
Rolling circle DNA amplification mechanism. Phage single-stranded circular DNA is converted to double-stranded replicative form (RF) by host enzymes after infection. P2 creates a nicking site at one strand of the RF. Then, DNA polymerase recognizes the nicked 3′ end and synthesizes the new DNA strand. P2 digests the new DNA strand again at the same specific recognition site. (Regenerated according to Sambrook (2001) [2] via Inkscape Project software, v.1.3.2.)

**Figure 2:**
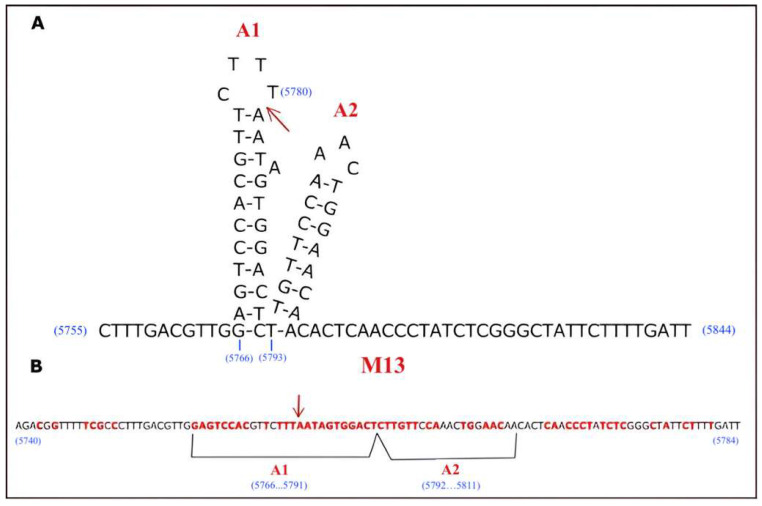
P2 recognition site in M13 filamentous bacteriophage genome (NC_003287.2). (**A**) A1 and A2 are two loop structures of the recognition site, and the arrow indicates the nicking site. (**B**) Linearized DNA sequence of recognition site. Red inked nucleotides represent the required sequences for recognition of P2 from previous studies [15,16].

**Figure 3:**
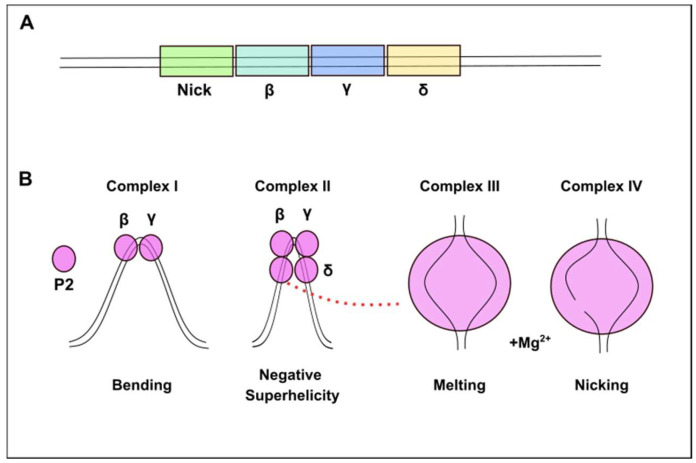
Conformational change in DNA created by the P2 during the nicking process. (**A**) Representing of the DNA sequence segments (β, γ and δ) on the recognition site. (**B**) When P2 binds to the recognition site, it bends the DNA to create negative superhelicity. P2 nicks one of the DNA strands after the melting step which is the denaturing of strands. (Regenerated according to Horiuchi K. (1997) [20] via Inkscape Project Software, v.1.3.2.)

M13 is a filamentous bacteriophage that carries a single-stranded DNA (ssDNA) molecule and has a non-integrative lysogenic life cycle and infects only male enterobacteria that carry F pilus (F episome, F+) [1,2]. While phage coat proteins are present on the phage particle, the functional proteins are synthesized in infected host cells by host enzymes from phage genomic DNA [3]. These functional proteins are present only in the bacterial host cell and play roles in the production and packaging of the newly generated phage particles. M13 infection starts with the binding of a fusion protein of a phage called P3 to the F episome of the host bacteria [2,4]. When the ssDNA genome of the phage (NC_003287.2) is transferred into the cell, RNA polymerase generates a primer from the transferred strand (+). The complementary strand (−) synthesizes from replication origin by DNA Polymerase III of the bacteria, which converts the ssDNA to a double-stranded DNA (dsDNA) form that is called replicative DNA form I (RFI DNA) [2,4,5]. Then, amplification of phage genomic DNA is achieved by a rolling circle amplification mechanism. One of the proteins of M13, *gene II* Protein as endonuclease (Gp2/P2) (Uniprot #P69547), recognizes the specific gene sequence at the f1 origin (f1 ori) and creates a nick on one strand of the (+) double-stranded replicative DNA form II (RFII DNA) form [1,2,3,4]. Host DNA polymerase recognizes this 3′ end of the nicked strand and initiates polymerization using template DNA. When the synthesis of the whole genomic DNA is completed, P2 cuts the strand again at the same specific nicking site (Figure 1) and produces new progeny (+) DNA strands [6]. Viral proteins of M13, which are called Protein 2, 5, and 10 (P2, P5, and P10), remain in the cytoplasm and take a role in viral genome replication, assembly, and packaging of the phage. Proteins 1, 4, and 11 (P1, P4, and P11) create a transport complex in the inner and outer membrane, while Proteins 6, 7, 8, 9 (P6, P7, P8, P9) and Protein 3 (P3) clump in the membrane before packaging [7]. The released progeny ssDNA is coated with Protein 5 (P5), and this complex transports to the membrane for an export complex that consists of P1, P11, and P4 with phage structure proteins and is then ejected from the bacterial cell without lysis of the host’s cell membrane [6,7,8,9,10,11]. P2 plays an important role in this life cycle of filamentous bacteriophages. Researchers have shown that the *gene II* defective phage mutants cannot convert the supercoiled double-strand replicative DNA form (RFI DNA) to the relaxed replicative DNA form (RFII DNA), indicating a nicking activity for P2 [12]. DNA sequences of many filamentous phages are 98% identical with each other [8,13,14]. Previous studies have shown that the specific sequence of recognition sites for P2 is localized in the f1 ori of DNA [1,6,12]. The nick site in the sequence 5′-CTTT/ATT-3′ is localized in the intergenic region of the phage genome between the 5781st and 5782nd nucleotides [15,16]. One of the strands is drawn to form a hairpin of two secondary structures, known as A1 and A2, in the f1 ori sequence (Figure 2) [1,12,17,18]. It has been suggested that P2 creates the loops by bending the DNA strands and then cutting them (Figure 3) [19,20]. P2 not only cuts the RF DNA but also seals the newly generated ssDNA, which can be further used as a template for replication or as genetic material for new phage particles [15].

Viruses take their potential power to gain the ability to infect new host populations. Viruses present major public health issues when they use humans as a host, like Human Immunodeficiency Virus (HIV) and severe acute respiratory syndrome (SARS) pandemics. Thus, it would be beneficial to use heuristic methods to detect emerging diseases before they spread rapidly [21]. Emerging viruses are those that have entered new populations of hosts, and they are generally evaluated by epidemiological or patient-based approaches. Current patient-based approaches are inappropriate, as they are mostly reactive rather than predictive [21]. Alternative methods that draw on the evolutionary ecology of viruses may be better able to forecast the appearance of viruses and prediction of virus emergence. However, working on metazoan viruses is a great challenge, and the development of new models is desirable. Much research has shown that bacteriophages are appropriate model organisms for virus emergence studies because the population parameters can easily be manipulated [21]. They have several advantages for the expansion of host range parameters such as population size, growth rate, and mutation rate [21]. To clarify the molecular mechanisms of cellular processes, multiple aspects of filamentous phage life have been used as model systems. Intensive research in this field has produced important quantitative data on M13 biology. Many of these studies on DNA replication, mRNA degradation, and mRNA processing in M13 phages were particularly useful [3]. The DNA replication mechanism of M13 phages can be used as a model for ssDNA virus amplification. According to this, nicking activity features of P2 can be important to establish the specific mechanisms for endonuclease-mediated DNA amplification systems. The latest study published about the P2 enzyme is more than 40 years old [6,22,23,24,25,26,27,28,29,30]. In this study, the nicking features of P2 have been determined in detail. While the exact restrictions of the minimal P2 recognition sequence which is in f1 ori were not given in previous studies and it included the other natural replication proteins in reaction, we used different parts of this recognition sequence as a substrate for the P2-GFP enzyme to investigate the region essential for nicking activity without any cooperative effect of the other proteins (Figure 2).

## 2. Results

### 2.1. Cloning of P2 Protein Gene and Protein Expression

The presence of recombinant plasmid DNA (p1GFP-P2) was verified by agarose gel electrophoresis according to size (7302 bp) and by Sanger sequencing. P2-GFP expression was monitored using GFP fluorescence as a reporter. Different fractions of cell culture samples at different conditions were collected according to a workflow (Figures S1 and S2). When the bacterial supernatants and pellets were directly imaged under blue light after centrifugation, fluorescent light indicated that P2-GFP was present in the cells. Therefore, cell pellets were lysed, and soluble/insoluble protein fractions were analyzed by 10% SDS-PAGE, 10% native PAGE, and Western blotting (Figure S3). The results showed that P2-GFP was present in the insoluble fraction. P2-GFP was only expressed in IPTG-induced samples and not in the non-induced samples. P2-GFP was best expressed with 0.5 mM IPTG at 37 °C for 5 h; on the native-PAGE gel, the brightness of the band for these conditions was greater than that of the bands for the other conditions (Figure S3).

### 2.2. Protein Purification and Validation

**Figure 4:**
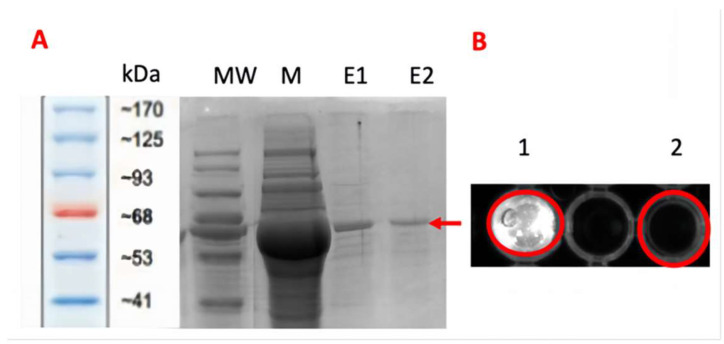
Purified protein validation. (**A**) The purification fractions of P2-GFP samples were separated by SDS-PAGE. Lanes E1 and E2 represent the elution fractions; lane M represents the crude extract of inclusion bodies. The red arrow indicates P2-GFP (~73 kDa). (**B**) Fluorescent analysis of the purified sample by ChemiDoc gel imaging system (Bio-Rad, South Granville, NSW, Australia). Well 1 contains the purified sample, and well 2 contains the test control.

The elution samples from IMAC-FPLC that were pooled and analyzed by SDS-PAGE are shown in Figure 4A. The His-tagged P2-GFP in the elution sample was verified by Western blotting. When the sample was analyzed under blue light by the ChemiDoc gel imaging system (Bio-Rad, South Granville, NSW, Australia), it showed fluorescence through the GFP part of the chimeric protein (P2-GFP), indicating the presence of P2-GFP in the elution fraction (Figure 4B). The protein concentration of the elution sample including P2-GFP was 32 ng/µL (4.4 µM). The GFP part of the chimeric protein was cleavage by tobacco etch virus (TEV) protease. As expected, the GFP fraction cleaved from the hybrid protein showed very intense florescent light, while P2 without GFP fraction did not (Figure S4).

### 2.3. P2 Activity Test

**Figure 5:**
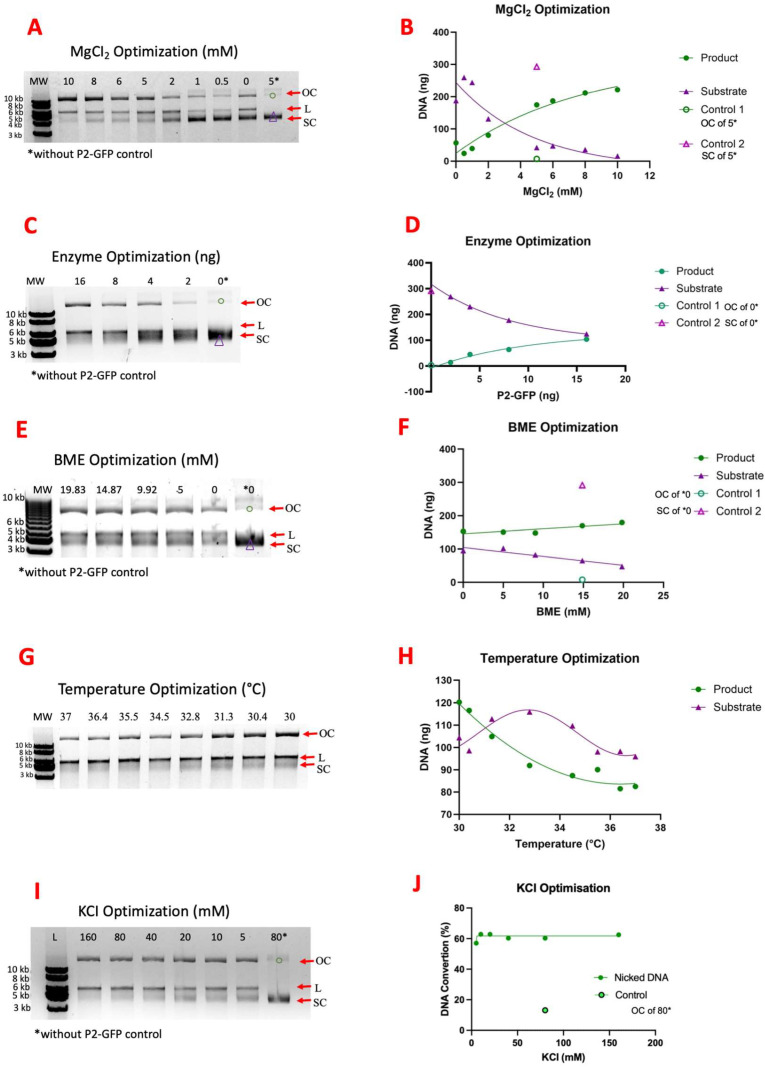
Agarose gel images of substrate–product analysis with different conditions (**A**,**C**,**E**,**G**,**I**) and quantitative band analysis (**B**,**D**,**F**,**H**,**J**). OC, L, and SC are open circular, linear, and supercoiled DNA, respectively. OC-DNA was used as a product, and SC-DNA was used as a substrate. “Control 1”, “Control”, and “Control 2” in graphics show OC-DNA and SC-DNA amounts of control reaction samples, respectively. MW is 1 kb DNA Ladder (Genemark, Taichung, Taiwan).

**Figure 6:**
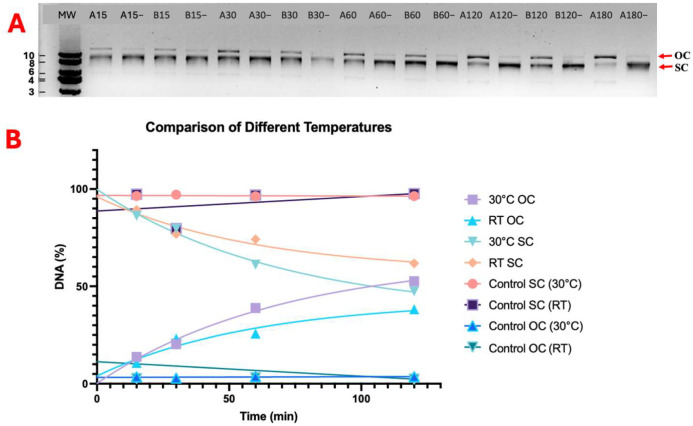
Temperature comparison of nicking reaction. (**A**) Agarose gel image of time course samples at 30 °C is labeled as “A” and room temperature (RT) is labeled as “B”. Time points are given from the 15th to 120th minute. Each time point is labeled with a number and temperature letter tag (A15 and B15 belong to 15th minute of the reaction sample at 30 °C and RT, respectively). Without P2-GFP enzyme control reactions represent “−” for each condition and time course. (**B**) Non-linear regression analysis of gel image. OC and SC represent open circular and supercoiled DNA, respectively. Controls show the band intensity of control samples. MW is 1 kb DNA Ladder, and each band is sized as kb.

The nicking reaction was monitored via the conversion of supercoiled DNA to open circular and linear forms. Plasmid DNA without P2-GFP enzyme treatment was used as a reference for topoisomeric conversion. When P2-GFP creates a nick on supercoiled (SC) DNA, the DNA is converted to an open circular (OC) form. The greatest nicking activity was observed at concentrations of 10 mM MgCl_2_, 80 mM KCl, and 10 mM β-mercaptoethanol (BME) at 30 °C (Figure 5). The comparison between the nicking activity at different temperatures is shown in Figure 5, and room temperature (20–25 °C) and 30 °C are shown in Figure 6. No difference in enzymatic activity was observed between the incubation at room temperature and that at 30 °C; the OC-DNA percentage increased at the same rate at both temperatures (Figure 6). Also, no difference in enzymatic activity was observed between the p1GFP plasmid DNA substrate and M13KE RF DNA as wild-type substrate (Figure S5). When the nicking activity of GFP-P2 and GFP cleavaged P2 were compared, they showed the same enzyme activity level (Figure S6).

### 2.4. Enzyme Kinetics

**Figure 7:**
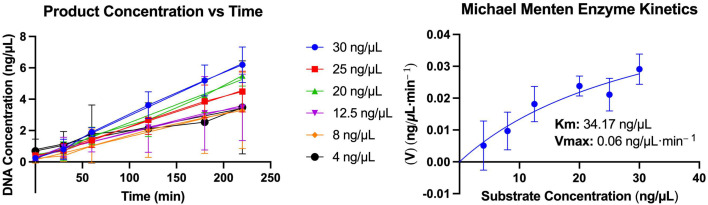
Kinetic analysis of P2-GFP. Open circular DNA intensity of different substrate concentrations and Michaelis–Menten equation for enzyme kinetics.

Michaelis–Menten equation analysis is given in Figure 7. The Vmax of the enzyme was 0.06 ng/µL·min^−1^, and the Km was 34.17 ng/µL (Figure 7).

### 2.5. Determination of Minimal Nicking Site

**Figure 8:**
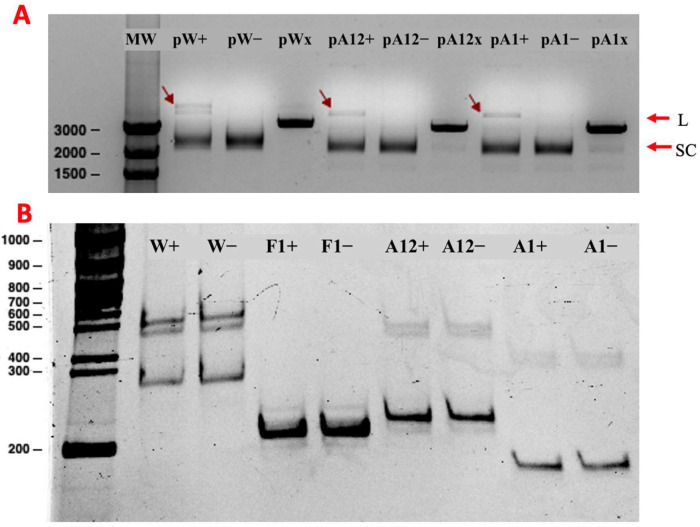
Gel images of different plasmid nicking products. (**A**) Agarose gel image of pW, pA12, and pA1 nicking reactions. “+” labeled plasmids represent P2-GFP-added reactions; “−label represents control samples (without P2-GFP). “x” represents the digested plasmid that was treated with the XhoI restriction enzyme as a conformational control of linearized DNA on the gel. P2-GFP cut both strands of supercoiled plasmids through carrying P2 recognition sites at both. Red arrows indicate linearized DNAs by P2-GFP. (**B**) DNA-PAGE image of linear DNA substrates amplified by PCR. While “+” labeled F1, W, A12, and A1 DNA fragments represent P2-added reactions, “−” represents control samples of reactions which include F1, W, A12, and A1 DNA fragments without P2-GFP. “F1+” and “F1−” belong to PCR products of f1 origin as a natural substrate. L and SC are linear and supercoiled DNA, respectively.

P2-GFP was able to cut both strands of pW, which carried the previously described whole recognition site, pA12 which included both loop-forming regions, and pA1 which only included a single loop where the cutting site was located (Figure 2 and Table S3). P2 was able to recognize and cut both strands of pA1, which carries the smallest fragment of the P2 recognition site in supercoiled form (Figure 8A). When the f1 origin DNA part (F1) and insert DNAs (W, A12, A1), which are cloned into pW, pA12, and pA1, were obtained by PCR and treated as linear form DNA substrate with P2-GFP, no cutting activity was observed (Figure 8B).

## 3. Discussion

In this study, we investigated the minimal DNA recognition site and in vitro enzymatic activity of the *gene II* product of the P2 nicking enzyme as an endonuclease of the M13 bacteriophage, which we obtained by cloning the P2 as a hybrid protein with GFP (P2-GFP) into *E. coli*. The most recent study about the features of P2 that we could find in the literature was published in 1987 [11]. Therefore, the current study is the first after almost 40 years to add more information about the recognition site of P2 by determining the minimal required DNA sequence for nicking activity and its biochemical activities under different conditions [6,22,23,24,25,26,27,28,29,30]. Also, P2 was expressed with GFP as the first hybrid protein composition of P2 in this study.

The first studies about P2 started with Geider K. and Meyer T. F. in 1979 [12]. In these first studies, P2 was isolated from M13 phage-infected *E. coli* cells to study the enzymatic activity of P2 in vitro. The reaction was performed with wild-type plasmid DNA (RF DNA) of M13 bacteriophages from infected cells, and enzyme activity was followed by the conformational change of SC-DNA to OC-DNA amount [12,31]. While they have shown the enzymatic reaction conditions for P2 nicking, they have used the other bacterial proteins that take roles in the natural replication of infected cells as the reaction mixture [12,31]. This may have led to a cooperative effect on the reaction. For this reason, we used P2-GFP protein without other proteins in the replication assays. When plasmid DNA, which is mainly in supercoiled form, was treated with P2, it was converted into an OC-DNA, indicating a nick in one of the strands created by P2. P2 needs a specific recognition site to cut DNA at one site. This recognition site and nicking signals were determined first by Horiuchi K. and his colleagues [6,11,20,32]. They showed that a more than 110 bp-sized DNA sequence was important for P2 binding, but results did not give exact restrictions for this recognition sequence. We used different parts of this recognition sequence, which included the natural recognition site (W), both loops (A12), and only the A1 loop (A1) sequences as a substrate DNA for the P2-GFP enzyme to investigate the region essential for nicking activity (Figure 2). Greenstein D. and Horiuchi K. (1987) showed that P2 was not able to cut the DNA if it had a linear DNA conformation [11]. Therefore, we cloned these truncated recognition site fragments (W, A12, and A1) into both strands of a cloning vector to obtain supercoiled plasmid DNA. P2-GFP’s nicking activity was monitored via conformational changes in the plasmid DNA. When generated plasmids were treated by P2-GFP, SC-DNA was converted into a linear DNA form (L-DNA), indicating the nicking of both strands. The nicking reaction was observed for all recombinant plasmids carrying truncated versions, including the smallest part (pA1), which is the loop where the nicking site is present. When these different DNA fragments were used as linear substrates for the nicking reaction, there were no differences between the test and control groups. This suggests that P2-GFP requires the supercoiled DNA structure for the nicking reaction, as previously reported for the P2 enzyme by Meyer and Geider (1979) and Greenstein D. and Horiuchi K. (1987) [11,12]. However, because the DNA fragments obtained by P2-GFP’s nicking activity can be very small, it may be possible that these fragments went undetected in the electrophoresis gels.

The best expression condition for P2-GFP was determined to be 0.5 mM IPTG induction with 5 h incubation at 37 °C. Although it was expected that using the GFP fusion tag would make the recombinant protein soluble, P2-GFP was observed in inclusion bodies. A high concentration of urea (8 M) was not sufficient to solubilize the inclusion bodies. However, the addition of thiourea and SDS enabled the solubilization of the inclusion bodies. This methodological information may help other researchers who try to produce recombinant protein expression with GFP and have difficulties with protein purification from the inclusion bodies. Enzymatic kinetics were analyzed by the Michaelis–Menten kinetic equation. We calculated the Km as 34.17 ng/µL and the Vmax as 0.06 ng/µL·min^−1^ for P2-GFP using gel images showing the conversion of SC-DNA to OC-DNA (Figure 7). However, the error rate may be high for making predictions from the measured intensity of plasmid DNA molecules from gels.

Single-stranded DNA (ssDNA) viruses are economically and medically important pathogens [33]. Some of them can cause infections in humans and animals [21,33]. Recent studies have shown that they can easily spread and are highly diverse genetically [21,34]. Many ssDNA viruses have the rolling circle-mediated replication mechanism that is initiated by a distinct virus-encoded endonuclease [21,34]. Members of the genus *Circovirus* in the family *Circoviridae,* which infect birds and mammals, have an ssDNA genome and a rolling circle replication initiator protein (Rep). Rep recognizes a stem-loop structure with a conserved nine bases in the loop, which is located between the 5′ ends of the two main open reading frames (ORFs) and initiates the replication of the viral genome. When a cell is infected, ssDNA converts to dsDNA by cellular DNA polymerase I. Rep binds to the loop structure of replication origin and cuts a nick in the (+) strand and a host polymerase extends the 3′ end. Replication continues according to a rolling circle replication mechanism [35,36]. This rolling circle replication strategy is very similar to some other viruses such as *Geminivirus*, *Nanovirus*, and bacterial plasmids in the *pT181* family [34]. Especially, creating the loop structure on genomic DNA from a 111 bp-sized recognition site and nicking on this structure mechanisms are very similar to M13 phage replication. While P2 nick between the 5′-TT/AA-3′ site of the loop, Rep nick between 5′-TT/AC-3′ [15,16,35]. Tobias Steinfeldt and his colleagues (2001) worked with the Rep protein of *Circovirus*, and they determined the minimal recognition sequence of Rep, like our aim for P2 [35]. Eric Delwart and Linlin Li (2012) worked on expanding the genetic diversity and host range of the *Circoviridae* viral family and other Reps, as a protein that can be able to create a nick site for initiating rolling circle mediated replication, encoding small circular ssDNA genomes [34]. They were obtaining information about candidate hosts by using some of the *rep*-bearing circular genome sequence similarities in this virus family [34]. Another ssDNA virus is the human pathogen *parvovirus B19*, which causes inflammatory and bone marrow diseases [37]. In the lifecycle of B19, its replication is again very similar to M13 bacteriophage replication: in both virus types, viral ssDNA converts into double-stranded DNA after infection of a host cell. In B19 replication, non-structural protein 1 (NS1) specifically binds to its recognition sequence in the replication origin and creates a nick in the DNA strand. Replication continues according to the rolling hairpin replication model [38].

All these ssDNA virus replication examples show that they share very similar molecular mechanisms in their life cycle. Determining the minimal recognition site of the P2 may help to deepen understanding of the other ssDNA viruses’ replication mechanisms and hosts by using similarities in this field for further studies. Current patient-based approaches for the evaluation of emerging viruses that have new populations of hosts are inappropriate [21]. There is a desire for alternative methods and models that draw on the evolutionary ecology of viruses to predict their emergence [21]. Much research has shown that bacteriophages are appropriate model organisms for these studies because of their features that can easily be manipulated, such as population size, growth rate, and mutation rate [21]. DNA replication, mRNA degradation, and mRNA processing studies on M13 phages as a model organism serve for producing quantitative data [3]. Also, obtaining detailed information for any ssDNA viruses can help to develop treatment strategies for the other pathogenic ones. For example, in recent years, researchers have been working on anti-sense oligonucleotides (ASOs), which are chemically modified and able to bind specific RNA sequence targets for the treatment of ssDNA virus-caused infections like *parvovirus B19* infections [37,39,40,41,42,43]. M13 infections in *E. coli* may be a practical model for the development of drugs against single-stranded viruses that cause infections in humans.

## 4. Materials and Methods

### 4.1. Cloning of the P2 Gene

The P2 expression vector was constructed using the ligation-independent cloning (LIC) N-terminal fusion plasmid, pET His6 GFP TEV LIC cloning vector (p1GFP) (Addgene Plasmid #29663), which carries the green fluorescent protein (GFP) gene and the 6× histidine tag at the N terminus of the protein and is expressed as His6X-GFP-TEV-P2 (P2-GFP). Vector DNA was digested with SspI Fast Digest Restriction Enzyme (#R0132S, Thermo Fisher Scientific, Waltham, MA, USA) and then purified via agarose gel electrophoresis using a Gel DNA Recovery Kit (Zymo-Research, Orange, CA, USA). The P2 gene (GeneID: 927328) of the M13 bacteriophage was amplified from M13KE replicative DNA form (pM13KE) (NEB #E8101S) via PCR using the following cycling conditions: initial denaturation at 95 °C for 1 min, followed by 35 cycles of 95 °C for 10 s, 64 °C for 15 s, and 72 °C for 12 s, and a final extension step at 72 °C for 5 min. To clone P2 into the LIC vector, amplification was performed using the following LIC-tagged (bold) primers: 5′-**TAC TTC CAA TCC AAT GCA** ATG ATT GAC ATG CTA GTT TTA CGA-3′ and 5′-**TTA TCC ACT TCC AAT GTT ATT A**TA TGC GAT TTT AAG AAC TGG CTC-3′. All PCR products were purified using a PCR Clean-Up Kit (GeneMark, Taichung, Taiwan), and their concentrations were measured spectrophotometrically (Nanodrop, Thermo Fisher Scientific, Waltham, MA, USA). The vector DNA and insert DNA were treated with dGTP and dCTP, respectively, and with LIC Qualified T4 DNA Polymerase (Novagen, Merck, San Jose, CA, USA), as explained in the enzyme protocol. The prepared recombinant DNA constructs were transformed into competent *E. coli* BL21 protease-deficient bacteria via the heat-shock method [44]. The transformed cells with recombinant construction were selected on Luria–Bertani (LB) agar containing 50 μg/mL kanamycin (KAN). The colonies were screened for the P2 gene via PCR using the same cloning primers. The presence of the P2 gene was verified by sequencing (Eurofins Scientific, Hamburgh, Germany).

### 4.2. Protein Expression

To express and purify the P2-GFP hybrid, a colony of recombinant bacteria was inoculated into 5 mL of LB medium containing 50 μg/mL of KAN, and the culture was incubated with shaking at 180 rpm overnight at 37 °C. This overnight culture was used to inoculate a 1:100 (v:v) ratio of a fresh culture of the same medium, and the fresh culture was incubated under the same conditions described above. When the OD_600_ of the culture reached 0.6, expression was induced in each culture by the addition of different concentrations of IPTG (0.1 mM, 0.5 mM, and 1 mM) at two different temperatures (20 °C and 37 °C) for different time courses (1, 2, 3, 4, 5, and 18 h). Cell pellets and supernatants were collected by centrifugation at 5000× *g* for 10 min. The cell pellets were resuspended in B-PER solution (Thermo Fisher Scientific, Waltham, MA, USA) for separation of soluble and insoluble protein fractions (inclusion bodies). The samples were stored at −20 °C until further purification was performed.

The expression levels of the P2-GFPs were analyzed using various techniques. First, all samples were directly imaged using blue light via an ORTE Gel Imaging System (TiBO, Istanbul, Türkiye). All samples were run on 10% native-PAGE, and the fluorescence of the P2-GFP molecules was imaged using a ChemiDoc gel imaging system (Bio-Rad, South Granville, NSW, Australia) [45]. The samples were analyzed via 10% SDS-PAGE by staining the gel with Coomassie R250 staining solution [46]. For specific detection of His-tagged P2 by Western blotting, samples were treated with a 1:1000 dilution of primer antibody (Rabbit pAb to 6XHis, Abcam, Cambridge, UK) and a 1:20,000 dilution of HRP-conjugated secondary antibody (Goat pAb IgG-HRP, Abcam, Cambridge, UK).

### 4.3. Solubilization of Inclusion Bodies

Since it was determined that P2-GFPs were expressed as inclusion bodies, soluble proteins were removed using B-PER solution (Thermo Fisher Scientific, Waltham, MA, USA) treatment and centrifugation. The inclusion body pellet was solubilized by the addition of freshly prepared inclusion body dissolving solution (2% SDS, 6 M urea, and 2 M thiourea) and incubated for 1 h at room temperature. The mixture was then centrifuged at 16,000× *g* for 15 min. The supernatant was collected, and each milliliter of sample buffer was exchanged with 14 mL of Tris buffer (20 mM Tris-HCl, pH: 7.4) via an Amicon Ultra-15 50K Centrifugal Filter Device (Millipore, Merck, San Jose, CA, USA) to remove denaturants. This step was performed four times. The concentrated and buffer-exchanged sample was stored at −20 °C until further purification was performed.

### 4.4. Protein Purification

Purification of P2-GFP was performed by immobilized metal affinity chromatography (IMAC) with a HisTrap HP column (Cytiva, Wilmington, Germany) and a fast protein liquid chromatography system (FPLC, ÄKTA Pure Protein Purification System, Cytiva, Wilmington, Germany). The column was equilibrated with a 5-column volume (CV) of Tris buffer at a 1 mL/min flow rate. The sample was loaded into the column with a 0.250 mL/min flow rate from a fulfilled 1 mL sample loop. The column was washed with 10 CV of wash buffer (20 mM Tris-HCl, 500 mM NaCl, 20 mM imidazole, pH: 7.4) with a 1 mL/min flow rate. Elution was performed with 20 CV of elution buffer (20 mM Tris-HCl, 500 mM NaCl, 400 mM imidazole, pH: 7.4) with a 0.25 mL/min flow rate, and peaks were collected according to the A_280_. Analysis was conducted using UNICORN 7.0 software. The protein elution fraction was buffer-exchanged with Tris buffer to remove residual imidazole and concentrated using an Amicon Ultra-15 50K Centrifugal Filter Device (Millipore, Merck, San Jose, CA, USA). Histidine-GFP fusion tag was removed by using *Tobacco etch virus* (TEV) protease ProTEV Plus (Promega, Madison, WI, USA) treatment. The mixture was loaded into nickel agarose beads (Ni-NTA, GoldBio, St. Louis, MO, USA), which were equilibrated with Tris buffer. The enzyme carries His-tag, therefore it was bound to the nickel matrix with cleavaged fusion tags. P2 without GFP was collected with flow-through in the Tris buffer. The bound sample was eluted with elution buffer as a control of removed GFP and TEV protease molecules.

### 4.5. Analysis of Purified P2-GFP

The protein concentration of the purified sample was determined via BCA Assay (Thermo Fisher Scientific, Waltham, MA, USA), and all samples were analyzed by 10% SDS-PAGE by staining the gel with Coomassie R250 staining solution [46]. For specific detection of His-tagged P2-GFP by Western blotting, samples were treated with a 1:1000 dilution of primer antibody (Rabbit pAb to 6XHis, Abcam, Cambridge, UK) and a 1:20,000 dilution of HRP-conjugated secondary antibody (Goat pAb IgG-HRP, Abcam, Cambridge, UK). Fluorescence was imaged using 200 µL of the purified sample placed into 96-well black-bottom plates (Corning, Glendale, AZ, USA) under blue epi-illumination with a standard filter in the ChemiDoc gel imaging system (Bio-Rad, South Granville, NSW, Australia). The final P2-GFP and P2 without GFP samples were stored at −20 °C in 20 mM Tris-HCl (pH: 7.4), 1 mM DTT, and 20% glycerol stock solution.

### 4.6. P2-GFP Activity Test

The nicking activity of P2-GFP was induced using 300 ng of p1GFP plasmid DNA or M13KE RF DNA (NEB) and 2 µM of P2-GFP enzyme in a 21 µL reaction volume. DNA was prepared separately with a final 1× KCl buffer (20 mM Tris-HCl pH: 8.5, 80 mM KCl) before adding it to the reaction. The concentrations of BME, MgCl_2_, KCl, and enzyme, incubation time, and temperature were optimized in 1× reaction buffer (20 mM Tris pH: 8.5, 9.10% sorbitol, 5 µg BSA) separately. Then, enzymatic reaction of P2-GFP was performed as given in Table 1. P2-GFP and P2, which were cleaved by TEV enzyme samples, were compared at optimized reaction conditions to determine the differences. Reactions were terminated by the addition of 60 mM EDTA, and 120 ng of total DNA from the reaction was analyzed via 1% agarose gel electrophoresis. The gel was imaged using the ChemiDoc gel imaging system (Bio-Rad, South Granville, NSW, Australia).

### 4.7. Determination of Minimal Recognition Sequence for P2 Nicking

#### 4.7.1. Cloning of PCR Products Containing Different Segments of the P2 Nicking Site

Different insert DNAs were prepared from the SARS-CoV-2 virus genome using designed primers that carry different parts of the P2 recognition sites (Table 2). cDNA conversion was performed by incubating SARS-CoV-2 virus particles, which are inactivated by gamma-irradiation (provided by Acibadem LabCell Laboratory) with the AMV reverse transcriptase enzyme (NEB, Ipswich, MA, USA), with random primers (Thermo Fisher Scientific, Waltham, MA, USA) at 42 °C for 1 h. Each insert was amplified from the cDNA by PCR under the conditions described in Tables S1 and S2. The PCR products were analyzed by agarose gel electrophoresis, and amplicons were purified from the gel using an EZNA Gel Elution Kit (Zymo-Research, Orange, CA, USA). Inserts were ligated into the pUCm-T vector using a TA Cloning Kit (Bio Basic, Singapore). The prepared recombinant DNA constructs, which included inserts W, A12, and A1 (pW, pA12, and pA1), were transformed into competent *E. coli* DH5α via the heat-shock method [44]. The transformed cells with recombinant construction were selected on LB agar containing 100 μg/mL ampicillin (AMP), 200 µg/mL X-Gal, and 1 mM IPTG by blue-white screening. The white colonies were screened for insert genes by PCR using A1 insert amplification primers. The presence of insert genes was verified by agarose gel electrophoresis. Plasmids were isolated from the transformant culture using a plasmid isolation kit (GeneMark, Taichung, Taiwan), and inserts were reverified by PCR.

#### 4.7.2. Nicking Reactions of Different Segments of the P2 Recognition Sequence

For nicking of linear DNA samples, the wild-type recognition site of P2 (f1 ori-F1) (191 bp) was amplified from the p1GFP plasmid via PCR using the following primers: 5′-AGG GTT CCG ATT TAG TGC TT-3′ and 5′-AAT CAA AAG AAT AGC CCG AGA TAG-3′. Plasmids in the supercoiled form, the f1 ori, and the insert DNAs as the linear forms of the recognition site, including different regions of the recognition site, were digested with the P2 enzyme (Table 1, Table 2 and Table S1) at room temperature for 2 h, and the reaction was terminated using 50 mM EDTA. In addition, plasmids were digested with a single-site cutting enzyme, XhoI, as a reference for linear DNA in electrophoresis. All samples were analyzed by agarose gel electrophoresis and 12% DNA-PAGE with 2× loading dye (90% DMSO, 0.5% EDTA, 0.1% xylene cyanole, and 0.1% bromophenol blue) [46].

### 4.8. Data Analysis

The band intensities from the agarose gel images were converted to quantitative values in ImageJ software (v.1.54) [47]. Each band’s intensity was compared to that of the other samples and controls. The percentage of product DNA conversion was calculated depending on the band intensity, and differences between samples were shown as the relative fold-change compared to the control. The data were converted into graphics using Prism software (v.10.1).

### 4.9. Enzyme Kinetics

The kinetics of the nicking reaction were investigated using 600, 300, 200, and 100 ng of substrate DNA in a 21 µL reaction volume. All kinetic experiments were carried out in triplicate. Samples were collected, and the reaction was stopped using 50 mM EDTA at 15, 30, 60, and 120 min time points. The samples were then analyzed by agarose gel electrophoresis. The gel images were converted into quantitative values. The enzyme kinetics were determined using the Michaelis–Menten equation in Prism software (v.10.1).

## Figures and Tables

**Table 1:** Nicking reaction mixture composition.

Reagent	Stock Concentration	Final Concentration	Volume (µL)
Reaction Buffer (20 mM Tris pH: 8.5, 9.10% sorbitol, 5 µg BSA)	3×	1×	7
BME (1/100)	143 mM	9.9 mM	1.45
P2-GFP/P2 Enzyme	2 µM	2 µM	1
Substrate DNA in 1× KCI Buffer (300 ng in 20 mM Tris-HCl pH: 8.5, 80 mM KCl)	-	300 ng	-
ddH_2_O	-	-	Up to 21-µL
**Preparation of Substrate DNA**			
DNA		300 ng	
KCI Buffer (20 mM Tris-HCl pH: 8.5, 80 mM KCl)	5×	1×	
**Reaction Conditions**			
Incubation at 30 °C for 30 min			

**Table 2:** List of inserts.

Insert Name	Description	Primer Pairs (Set)	Product Size (bp)
Insert W	dsDNA: SARS_CoV19 N1 site amplification with “whole” part recognition site included primers	N1P2 Fw/Std1 P2 Rv	264
Insert A12	dsDNA: SARS_CoV19 N1 site amplification with “A1 and A2 loop” part recognition site included primers	N1P2 A12 Fw/Std 1 P2 A12 Rv	208
Insert A1	dsDNA: SARS_CoV19 N1 site amplification with “A1 loop” part recognition site included primers	N1P2 A1 Fw/Std 1 P2 A1 Rv	158

## Data Availability

Data are contained within the article.

## Supplementary Materials

The following supporting information can be downloaded at: https://www.mdpi.com/article/10.3390/ijms26020789/s1.

## References

[1] Suggs S.V., Ray D.S. (1979). Nucleotide Sequence of the Origin for Bacteriophage M13 DNA Replication. Cold Spring Harb. Symp. Quant. Biol..

[2] Sambrook J.F., Russell D. (2001). M13 Bacteriophages. Molecular Cloning: A Laboratory Manual.

[3] Smeal S.W., Schmitt M.A., Pereira R.R., Prasad A., Fisk J.D. (2017). Simulation of the M13 Life Cycle II: Investigation of the Control Mechanisms of M13 Infection and Establishment of the Carrier State. Virology.

[4] Brasino M. (2016). Engineering of Filamentous Bacteriophage for Protein Sensing. Ph.D. Thesis.

[5] Russel M. (1995). Moving through the Membrane with Filamentous Phages. Trends Microbiol..

[6] Dotto G., Horiuchi K., Zinder N. (1984). The Functional Origin of Bacteriophage F1 DNA Replication. Its Signals and Domains. J. Mol. Biol..

[7] Smeal S.W., Schmitt M.A., Rodrigues R., Prasad A., Fisk J.D. (2017). Simulation of the M13 Life Cycle I: Assembly of a Genetically-Structured Deterministic Chemical Kinetic Simulation. Virology.

[8] Rakonjac J., Russel M., Khanum S., Brooke S.J., Raji M. (2017). Filamentous Phage: Structure and Biology. Recombinant Antibodies for Infectious Diseases. Advances in Experimental Medicine and Biology.

[9] Slonczewski J., Foster J. (2017). Viral Molecular Biology. Microbiology an Evolving Science.

[10] Dotto G.P., Enea V., Zinder N.D. (1981). Gene II of Phage F1: Its Functions and Its Products. Proc. Natl. Acad. Sci. USA.

[11] Greenstein D., Horiuchi K. (1987). Interaction between the Replication Origin and the Initiator Protein of the Filamentous Phage F1. Binding Occurs in Two Steps. J. Mol. Biol..

[12] Geider K., Meyer T. (1979). Gene-II Protein of Bacteriophage Fd in Enzymatic Replication of Viral Duplex DNA. Cold Spring Harb. Symp. Quant. Biol..

[13] Morag O., Sgourakis G.N., Abramov G., Goldbourt A., Ghose R. (2018). Filamentous Bacteriophage Viruses: Preparation, Magic-Angle Spinning Solid-State NMR Experiments, and Structure Determination. Protein NMR.

[14] Azzazy H.M.E., Highsmith W.E. (2002). Phage Display Technology: Clinical Applications and Recent Innovations. Clin. Biochem..

[15] Harth G., Bäumel I., Meyer T.F., Geider K. (1981). Bacteriophage Fd Gene-2 Protein: Processing of Phage Fd Viral Straands Replicated by Phage T7 Enzymes. Eur. J. Biochem.

[16] Meyer T., Geider K., Kurz C., Schaller H. (1979). Cleavage Site of Bacteriophage Fd Gene II-Protein in the Origin of Viral Strand Replication. Nature.

[17] Peeters B.P.H., Schoenmakers J.G.G., Konings R.N.H. (1987). Comparison of the DNA Sequences Involved in Replication and Packaging of the Filamentous Phages IKe and Ff (M13, Fd, and Fl). DNA.

[18] Baas P.D., Jansz H. (1988). Single-Stranded DNA Phage Origins. Curr. Top. Microbiol. Immunol..

[19] Higashitani N., Higashitani A., Guan Z.W., Horiuchi K. (1996). Recognition Mechanisms of the Minus-Strand Origin of Phage F1 by Escherichia Coli RNA Polymerase. Genes Cells.

[20] Horiuchi K. (1997). Initiation Mechanisms in Replication of Filamentous Phage DNA. Genes Cells.

[21] Dennehy J.J. (2009). Bacteriophages as Model Organisms for Virus Emergence Research. Trends Microbiol..

[22] Geider K., Bäumel I., Meyer T. (1982). Intermediate Stages in Enzymatic Replication of Bacteriophage Fd Duplex DNA. J. Biol. Chem..

[23] Horiuchi K., Zinder N. (1976). Origin and Direction of Synthesis of Bacteriophage Fl DNA. Proc. Natl. Acad. Sci. USA.

[24] Cleary J.M., Ray D. (1981). Deletion Analysis of the Cloned Replication Origin Region from Bacteriophage M13. J. Virol..

[25] Johnston S., Ray D. (1984). Interference between M13 and OriM13 Plasmids Is Mediated by a Replication Enhancer Sequence near the Viral Strand Origin. J. Mol. Biol..

[26] Meyer T.F., Geider K. (1979). Bacteriophage Fd Gene II-Protein. II. Specific Cleavage and Relaxation of Supercoiled RF from Filamentous Phages. J. Biol. Chem..

[27] Meyer T., Geider K. (1982). Enzymatic Synthesis of Bacteriophage Fd Viral DNA. Nature.

[28] Lin N., Pratt D. (1974). Bacteriophage M 13 Gene 2 Protein: Increasing Its Yield in Infected Cells, and Identification and Localization. Virology.

[29] Schaller H., Uhlmann A., Geider K. (1976). A DNA Fragment from the Origin of Single-Strand to Double-Strand DNA Replication of Bacteriophage Fd. Proc. Natl. Acad. Sci. USA.

[30] Dotto G., Enea V., Zinder N. (1981). Functional Analysis of Bacteriophage F1 Intergenic Region. Virology.

[31] Meyer T.F., Geider K. (1981). Cloning of the Bacteriophage Fd Gene 2 and Construction of a Plasmid Dependent on Fd Gene 2 Protein. Proc. Natl. Acad. Sci. USA.

[32] Dotto G., Horiuchi K., Jakes K., Zinder N. (1982). Replication Origin of Bacteriophage F1. Two Signals Required for Its Function. J. Mol. Biol..

[33] Krupovic M., Koonin E.V. (2014). Evolution of Eukaryotic Single-Stranded DNA Viruses of the Bidnaviridae Family from Genes of Four Other Groups of Widely Different Viruses. Sci. Rep..

[34] Delwart E., Li L. (2012). Rapidly Expanding Genetic Diversity and Host Range of the Circoviridae Viral Family and Other Rep Encoding Small Circular SsDNA Genomes. Virus Res..

[35] Steinfeldt T., Finsterbusch T., Mankertz A. (2001). Rep and Rep’ Protein of Porcine Circovirus Type 1 Bind to the Origin of Replication in Vitro. Virology.

[36] Faurez F., Dory D., Grasland B., Jestin A. (2009). Replication of Porcine Circoviruses. Virol. J..

[37] Spurgers K.B., Sharkey C.M., Warfield K.L., Bavari S. (2008). Oligonucleotide Antiviral Therapeutics: Antisense and RNA Interference for Highly Pathogenic RNA Viruses. Antiviral. Res..

[38] Ganaie S.S., Qiu J. (2018). Recent Advances in Replication and Infection of Human Parvovirus B19. Front. Cell. Infect. Microbiol..

[39] Yuen M.F., Heo J., Jang J.W., Yoon J.H., Kweon Y.O., Park S.J., Tami Y., You S., Yates P., Tao Y. (2021). Safety, Tolerability and Antiviral Activity of the Antisense Oligonucleotide Bepirovirsen in Patients with Chronic Hepatitis B: A Phase 2 Randomized Controlled Trial. Nat. Med..

[40] Quemener A.M., Galibert M.D. (2022). Antisense Oligonucleotide: A Promising Therapeutic Option to Beat COVID-19. Wiley Interdiscip. Rev. RNA.

[41] Suzuki Y., Ishimoto T., Fujita S., Kiryu S., Wada M., Akatsuka T., Saito M., Kawano M. (2020). Antimicrobial Antisense RNA Delivery to F-Pili Producing Multidrug-Resistant Bacteria via a Genetically Engineered Bacteriophage. Biochem. Biophys. Res. Commun..

[42] Guan W., Huang Q., Cheng F., Qiu J. (2011). Internal Polyadenylation of the Parvovirus B19 Precursor MRNA Is Regulated by Alternative Splicing. J. Biol. Chem..

[43] Ly C.V., Miller T.M. (2018). Emerging Antisense Oligonucleotide and Viral Therapies for Amyotrophic Lateral Sclerosis. Curr. Opin. Neurol..

[44] Chung C.T., Niemela S.L., Miller R.H. (1989). One-Step Preparation of Competent Escherichia Coli: Transformation and Storage of Bacterial Cells in the Same Solution. Proc. Natl. Acad. Sci. USA.

[45] Arndt C., Koristka S., Bartsch H., Bachmann M., Walker J.M., Kurien B.T., Scofield R.H. (2012). Native Polyacrylamide Gels. Methods in Molecular Biology.

[46] Green M.R., Sambrook J. (2020). Polyacrylamide Gel Electrophoresis. Cold Spring Harb. Protoc..

[47] Abramoff M.D., Magalhaes P.J., Ram S.J. (2004). Image Processing with ImageJ. Biophotonics Int..

