# Supplementary material for: Nicking Activity of M13 Bacteriophage Protein 2
Source: Int J Mol Sci. 2025 Jan 18;26(2):789. doi: 10.3390/ijms26020789 (PMC11765958; doi:10.3390/ijms26020789)
Supplement: Supplementary file 1 [file ijms-26-00789-s001.zip › ijms-3345397-supplementary.pdf]

Supplementary Materials

Supplementary Tables

Table S1: PCR conditions for inserts.

Initial Denaturation	95°C	3 minutes
*Denaturing	95°C	30 seconds
*Annealing	**56/61°C	20 seconds
*Extension	72°C	30 seconds
Final Extension	72°C	5 minutes
Final Temperature	4°C	∞

*35 cycles

**56°C for A12 and A1 inserts; 61°C for W insert.

Table S2: List of insert primers specific to Sars Cov-19

Primer Name	Nucleotide Sequence (5'-3')	Size (bp)	GC%	Tm (°C)
***N1P2 Fw**	**CTTTGACGTTGGAGTCCACGTTCTTTAATAGTGGACTCTTGTTCCAAACTGGAACAACACTCAACCCTATCTCGGTCTATTCTTTTGATT**GACCCCAAAATCAGCGAAAT	110	41.8	71
***N1P2-A1 Fw**	**CGTTGGAGTCCACGTTCTTTAATAGTGGACTCTACAC**GACCCCAAAATCAGCGAAAT	57	45.6	68.6
**N1P2-A12 Fw**	**CTTTGACGTTGGAGTCCACGTTCTTTAATAGTGGACTCTTGTTCCAAACTGGAACAACACTC**GACCCCAAAATCAGCGAAAT	82	43.9	70.6
**Std1 P2 Rv**	**CTTTGACGTTGGAGTCCACGTTCTTTAATAGTGGACTCTTGTTCCAAACTGGAACAACACTCAACCCTATCTCGGTCTATTCTTTTGATT**TGCGTTCTCCATTCTGGTTA	110	41.8	70.8
**Std1 P2-A1 Rv**	**CGTTGGAGTCCACGTTCTTTAATAGTGGACTCTACAC**TGCGTTCTCCATTCTGGTTA	57	45.6	68.1
**Std 1 P2-A12 Rv**	**CTTTGACGTTGGAGTCCACGTTCTTTAATAGTGGACTCTTGTTCCAAACTGGAACAACACTC**TGCGTTCTCCATTCTGGTTA	82	43.9	70.3

bold letters show P2 digested parts at the recognition site

*N1: 2019- nCoV_N1 Forward Primer derived

2

Table S3: List of insert sequences of different parts of P2 recognition site and f1 origin part

Sequence Name	Sequence (5'-3')	Size (bp)
**W**	CTT TGA CGT TGG AGT CCA CGT TCT TTA ATA GTG GAC TCT TGT TCC AAA CTG GAA CAA CAC TCA ACC CTA TCT CGG TCT ATT CTT TTG ATT GAC CCC AAA ATC AGC GAA ATG CAC CCC GCA TTA CGT TTG GTG GAC CCT CAG ATT CAA CTG GCA GAT TGG TCT TAC CTC TTG CGT TTA GTT TTC TTA TCT GGC TCT ATC CCA ACT CAC AAC AAG GTC AAA CCT TGT TCT CAG GTG ATA ATT TCT TGC ACC TGA GGT TGC AGT TTC	264
**A12**	CTT TGA CGT TGG AGT CCA CGT TCT TTA ATA GTG GAC TCT TGT TCC AAA CTG GAA CAA CAC TCG ACC CCA AAA TCA GCG AAA TGC ACC CCG CAT TAC GTT TGG TGG ACC CTC AGA TTC AAC TGG CAG ATT GGT CTT ACC TCT TGC GTC TCA CAA CAA GGT CAA ACC TTG TTC TCA GGT GAT AAT TTC TTG CAC CTG AGG TTG CAG TTT C	208
**A1**	CGT TGG AGT CCA CGT TCT TTA ATA GTG GAC TCT ACA CGA CCC CAA AAT CAG CGA AAT GCA CCC CGC ATT ACG TTT GGT GGA CCC TCA GAT TCA ACT GGC AGA TTG GTC TTA CCT CTT GCG TCA CAT CTC AGG TGA TAA TTT CTT GCA CCT GAG GTT GC	158
**F1**	AGG GTT CCG ATT TAG TGC TTT ACG GCA CCT CGA CCC CAA AAA ACT TGA TTA GGG TGA TGG TTC ACG TAG TGG GCC ATC GCC CTG ATA GAC GGT TTT TCG CCC TTT GAC GTT GGA GTC CAC GTT CTT TAA TAG TGG ACT CTT GTT CCA AAC TGG AAC AAC ACT CAA CCC TAT CTC GGT CTA TTC TTT TGA TT	191

Supplementary Figures

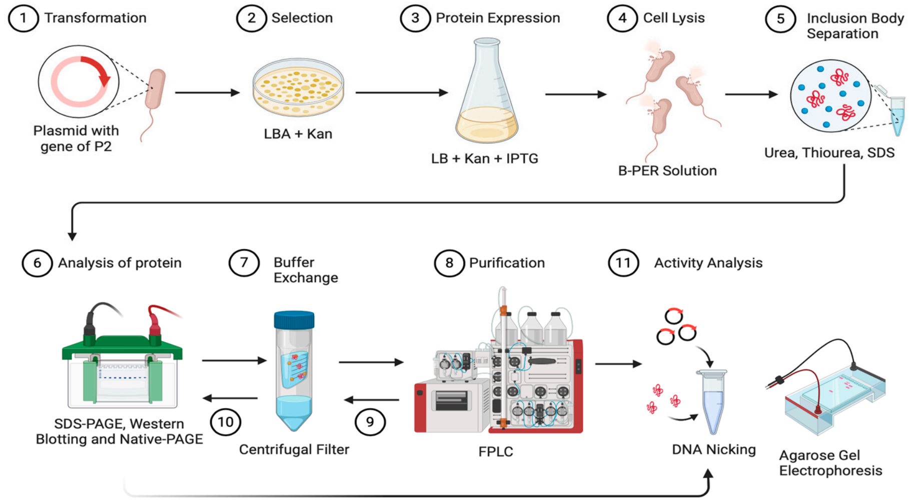

Figure S1: P2 cloning, expression, purification, and analysis workflow. After the transformation of a recombinant plasmid, P2-GFP was expressed, and cell lysate was analyzed by using SDS-PAGE, Native-PAGE, and Western Blotting methods. P2-GFP was purified from the collected sample mixture by FPLC and samples were analyzed again by the same methods.

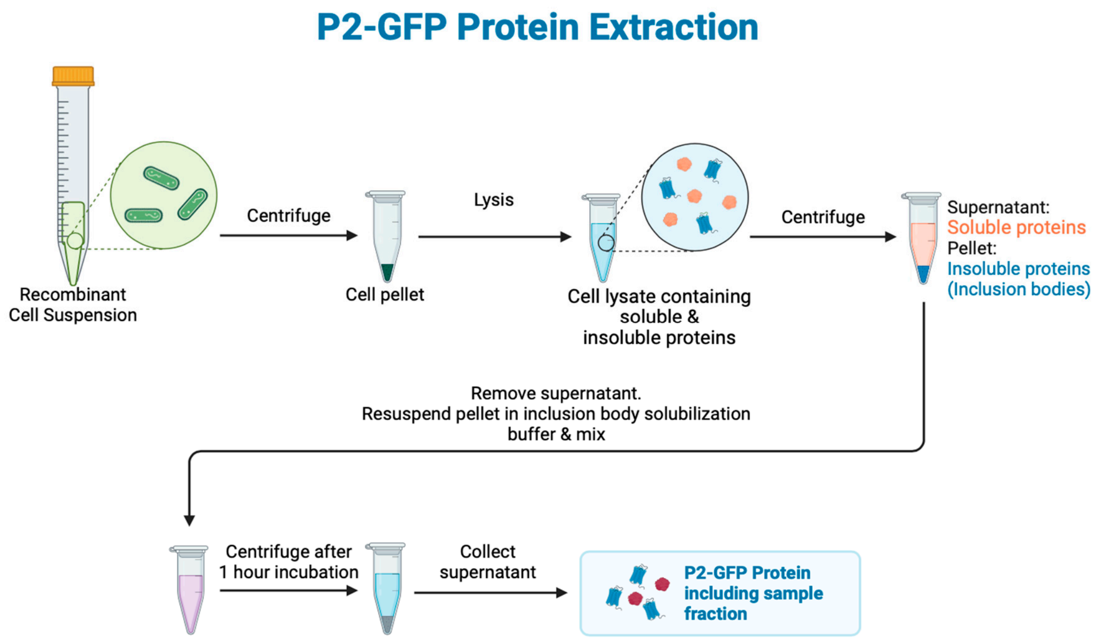

Figure S2: P2-GFP protein extraction workflow. It was performed by adding lysis solution to cell pellets to remove the soluble protein fraction from the inclusion bodies. The inclusion body pellet was resuspended in a freshly prepared inclusion body resolving solution for 1 h at room temperature. The mixture was centrifuged, and the supernatant, including the dissolved P2-GFPs, was collected. [Figure was created in BioRender.com].

4

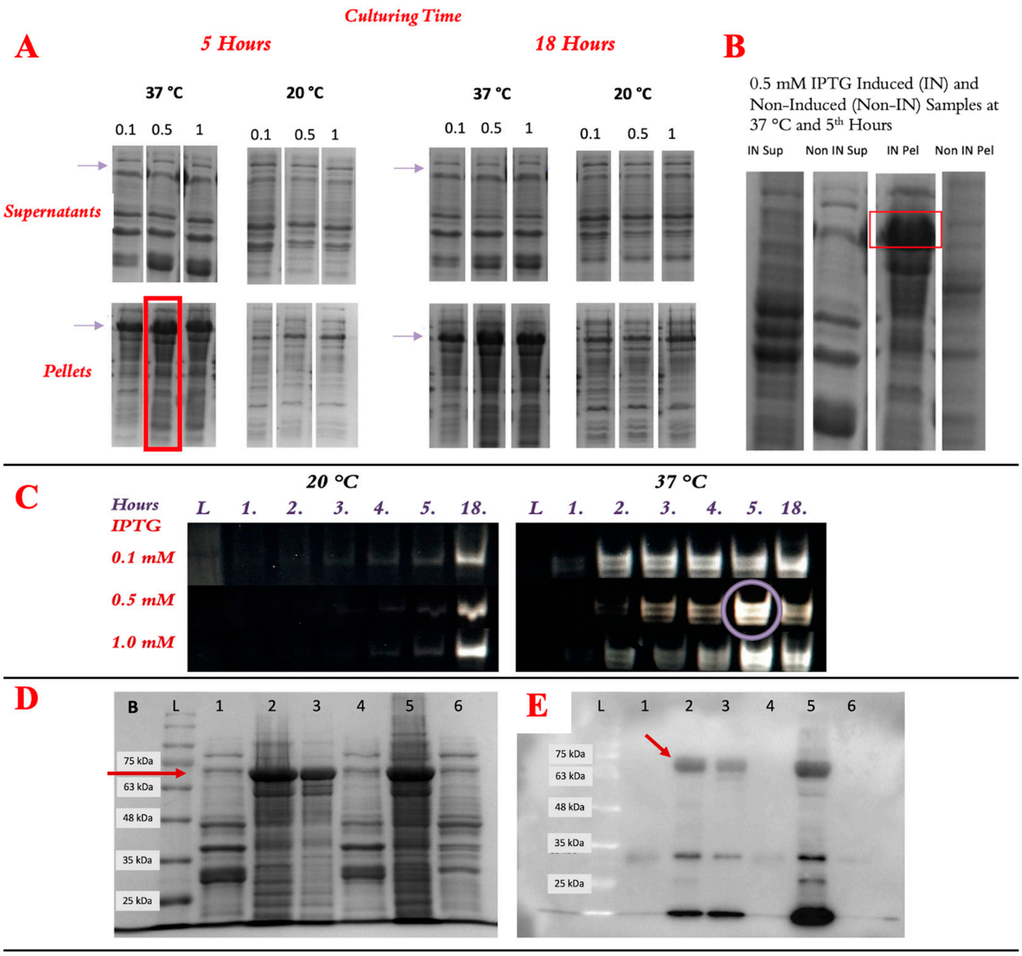

Figure S3: Protein Analysis Gel Images. **(A)** P2-GFP (indicated with an arrow) was observed in a cell pellet and 37 °C with 0.5 mM IPTG inducing for 5h incubation was more intense than others (red box). **(B)** Comparing 0.5 mM IPTG induced and non-induced protein samples at 37 °C and 5 h incubation. P2-GFP (red box) is only seen in induced samples. **(C)** The Native-PAGE gel images of supernatant samples of B-PER treated cell pellets (soluble protein fraction). The brightness of the band depends on GFP expression for optimal conditions that were observed more brighter than other conditions (in a circle). **(D)** SDS-PAGE gel of western blotting with Coomassie brilliant blue. L is protein marker; 1 and 4 are the supernatant sample belongs to 37 °C with 0.5 mM IPTG induced for 5h and 18h; 2 and 5 are the pellet sample belongs to 37 °C with 0.5 mM IPTG induced for 5h and 18h; 3 is the inclusion body solved sample belongs to 37 °C with 0.5 mM IPTG induced for 5h; 6 is the supernatant sample belongs to 20 °C with 0.5 mM IPTG induced for 18h incubations respectively. **(E)** Western Blotting Image. P2-GFP is indicated with an arrow.

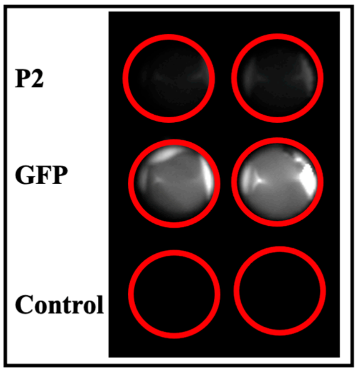

Figure S4: Fluorescent analysis of TEV cleavage P2 (P2) and removed GFP (GFP). The removed GFP sample gives more bright fluorescent light than the P2-GFP sample. Control includes nuclease-free water.

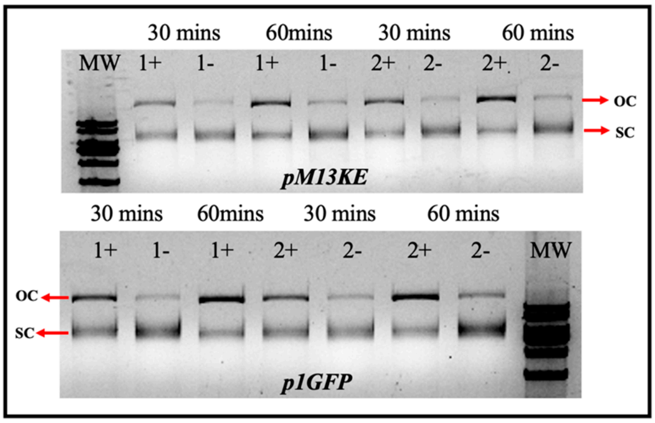

Figure S5: Agarose gel images of *pM13KE* and *p1GFP* nicking reaction samples at different time courses. Duplicate reactions with and without P2-GFP enzyme samples were labeled as “1+”, “2+”; and “1-“, and”-2” respectively. **OC** and **SC** are open circular, and supercoiled DNA, respectively.

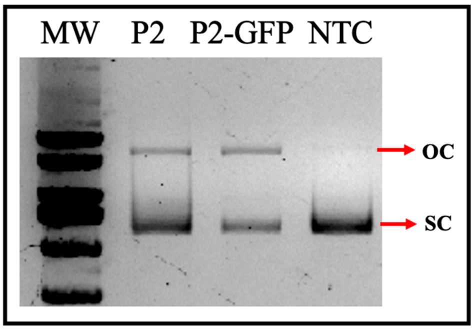

N

Figure S6: Agarose gel image of P2 and P2-GFP nicking reaction samples. Nicking reaction samples of P2-GFP and GFP fusion tag removed P2 (P2) gives the same product as open circular DNA (OC). SC is supercoiled DNA as a substrate. Control (NTC) includes only substate in the reaction without enzyme.

6
